# Supplementary material for: Oncotree2vec — a method for embedding and clustering of tumor mutation trees
Source: Bioinformatics. 2024 Jun 28;40(Suppl 1):i180–8. doi: 10.1093/bioinformatics/btae214 (PMC11211817; doi:10.1093/bioinformatics/btae214)
Supplement: btae214_Supplementary_Data [file btae214_supplementary_data.pdf]

oncotree2vec – A method for embedding and clustering of tumor mutation trees

Monica-Andreea Baciu-Drăgan[1,2] and Niko Beerenwinkel[1,2,*]

[1] Department of Biosystems Science and Engineering, ETH Zürich, Schanzenstrasse 44, 4056, Basel, Switzerland
[2] SIB Swiss Institute of Bioinformatics, Schanzenstrasse 44, 4056, Basel, Switzerland

[*]Corresponding author. E-mail: niko.beerenwinkel@bsse.ethz.ch

Supplementary information

Supplementary Table 1: Overview of the mutation tree cohorts used in the experiments. For the experiment with real trees with different modes of evolution we are interested only in the tree structures, therefore reporting the number of mutations is not applicable.

Experiment	Number of samples	Total number of clones	Average number of nodes per sample	Average node degree	Number of mutations
Synthetic dataset I	315	2,331	7.4	1.72	108
Synthetic dataset II	52	2,600	50	1.96	1,325
Synthetic dataset III	26	208	8	1.62	26
Real trees with different modes of evolution	43	667	15.5	1.87	NA
AML mutation trees	123	641	5.2	1.8	31

Supplementary Table 2: Parameters used in the experiments described in Section 3 of the main paper. The use of the parameters is described in Section 2.2. For the two experiments with not applicable kernel size (NA) we discard the tree vocabulary words corresponding to different neighborhoods, by the design of the experiment. We used WL kernel size 9, equal to the longest path from root to leaves in the cohort, for the first synthetic dataset containing 16 groups of trees, in order to ensure that we match entire tree structures, since the dataset was designed such that the trees from the same group share the same tree structure. The vocabulary categories not listed in the table were discarded.

Experiment	Max WL kernel size	Vocabulary categories	Augmentation amounts	Embedding size (δ)	Number of training iterations until convergence	Vocabulary size (number of words)	CPU time on a desktop PC
Synthetic dataset I	9	Neighborhoods<br>Tree structures	1<br>1	128	7,000	92,784	2h31
Synthetic dataset II	NA	Individual nodes	1	64	200	5,200	0m46
Synthetic dataset III – oncotree2vec WL kernel size 0	NA	Root-child<br>Direct edges<br>Same path<br>Mutual exclusivity	1<br>1<br>1<br>1	32	600	60	0m51
Synthetic dataset III – oncotree2vec WL kernel size 1	NA	Neighborhoods<br>Root-child<br>Direct edges<br>Same path<br>Mutual exclusivity	1<br>5<br>5<br>5<br>5	32	600	740	1m
Real trees with different modes of evolution	3	Tree structures	5	64	500	4,906	3m34
AML mutation trees	3	Neighborhoods<br>Individual nodes<br>Root-child<br>Direct edges<br>Same path<br>Mutual exclusivity	1<br>5<br>20<br>10<br>10<br>10	128	1,500	33,920	1h45
Non-small-cell lung cancer – mutation trees	0	Root-child<br>Direct edges<br>Same path<br>Mutual exclusivity	1<br>1<br>1<br>1	128	1,000	1356	3m13

Supplementary Table 3: Clusters in the AML mutation tree cohort, sorted by cluster size. The mean survival time is indicated for clusters of 4 samples or more. The cluster ids correspond to the ones in Suppl. Fig. 11. The colored rows correspond to groups of clusters (the blue clusters vs the yellow one) with significant difference in the survival curves according to the pairwise log-rank test; the result is confirmed by the values of the median survival time. The NA values indicate that the median survival could not be computed because the survival data did not drop below 50% at the end of the available data (see also the Kaplan Meier curves in Suppl. Fig. 11E).

Number of samples	Shared mutations	Median survival time (number of months)	Cluster id
18	DNMT3A primary mutation<br>subcluster of DNMT3A, NRAS co-occurrence<br>subcluster of DNMT3A, NPM1 co-occurrence<br>subcluster of DNMT3A, FLT3-ITD co-occurrence	17.9	0
16	IDH2 primary mutation<br>subcluster of IDH2, SRSF2 co-occurrence<br>subcluster of IDH2, NPM1 co-occurrence	55.3	1
11	TET2 primary mutation<br>subcluster of TET2, NPM1 co-occurrence	11.2	2
9	NRAS primary mutation	12.9	3
7	TP53 primary mutation	9.3	4
6	WT1 primary mutation	12.65	5
6	SF3B1 primary mutation	13.9	6
5	IDH1 primary mutation	16.7	7
5	NPM1 primary mutation<br>PTPN11, KRAS clonal exclusivity	24.9	8
4	FLT3-ITD primary mutation	9.1	9
4	SRSF2 primary mutation	23.57	10
4	NPM1 primary mutation	NA	11
4	FLT3 primary mutation<br>FLT3, NRAS clonal exclusivity	NA	12
3	TP53, DNMT3A co-occurrence	-	-
3	IDH2, DNMT3A, SRSF2 co-occurrence	-	-
2	PTPN11, NPM1 co-occurrence (identical samples)	-	-
2	EZH2, ASXL1, RUX1	-	-
2	IDH2, NPM1, SRSF2 co-occurrence	-	-
2	DNMT3A, KRAS, NPM1, FLT3 co-occurrence	-	-
2	DNMT3A, SF3B1, FLT3, RUNX1 co-occurrence	-	-

Algorithm 1 ComputeTreeVocabulary($G\{N, E, L\}, max\_wlk$)

```
input: tree G{N, E} with the corresponding node labels L and the maximum Weisfeiler-Lehman kernel size max_wlk.
output: the set of words V that form the vocabulary for the input tree.

V ⇐ {}
for each node ∈ G do
    if node = Root(G) then                                        ▷ Add root-child relations.
        word ⇐ Root(G).id ⊕ node.id
        V ⇐ V ∪ word
    end if
    for degree ∈ {0..max_wlk} do                                  ▷ Add neighborhoods of degree up to max_wlk.
        if degree(n) ≠ 1 then        ▷ This condition is applied if the vocabulary also includes direct edges.
            word ← node.id ⊕ sort(GetNeighborhood(node, G, degree))   ▷ Sort neighbors by node id.
            V ⇐ V ∪ word
        end if
    end for
    path_nodes ← GetPathToRoot(node, G)                           ▷ Add non-adjacent same path pairs.
    for idx ∈ {1..path.length − 1} do                             ▷ Exclude root and direct ancestor.
        word ← path_nodes[idx] ⊕ node.id
        V ⇐ V ∪ word
    end for
    V ⇐ V ∪ path_nodes[−1].id ⊕ node.id                           ▷ Add direct-edge.
end for

paths ← GetAllRootLeafPaths(G)
path_pairs ← Combinations(paths, 2)                               ▷ For all pairs on root-leaf paths.
for each path_pair ∈ path_pairs do                                ▷ Add mutually exclusive nodes.
    node_set1 ← path_pair[0] \ path_pair[1]                       ▷ Extract the branching nodes.
    node_set2 ← path_pair[1] \ path_pair[0]
    node_pairs ← {(n1, n2)|n1 ∈ node_set1 & n2 ∈ node_set2}
    for each node_pair ∈ node_pairs do
        node_pair ← sort(node_pair[0].id, node_pair[1].id)         ▷ Unique order, sorted by node id.
        word ← node_pair[0] ⊕ node_pair[1]
        V ⇐ V ∪ word
    end for
end for
                                                                  ▷ Add unlabeled tree structures.
for each node ∈ G do   ▷ Relabel the nodes such that Root has label 0 and internal nodes have label 1. Note: labels 0 and 1 were previously kept as reserved labels and not used in the tree labeling.
    if node = Root(G) then
        node.id ⇐ 0
    else[node ≠ Root(G)]
        node.id ⇐ 1
    end if
end for
for each node ∈ G do
    for degree ∈ {1..max_wlk} do                                  ▷ Discard individual nodes.
        V ⇐ V ∪ node.id ⊕ sort(GetNeighborhood(node, G, degree))
    end for
end for

return V
```

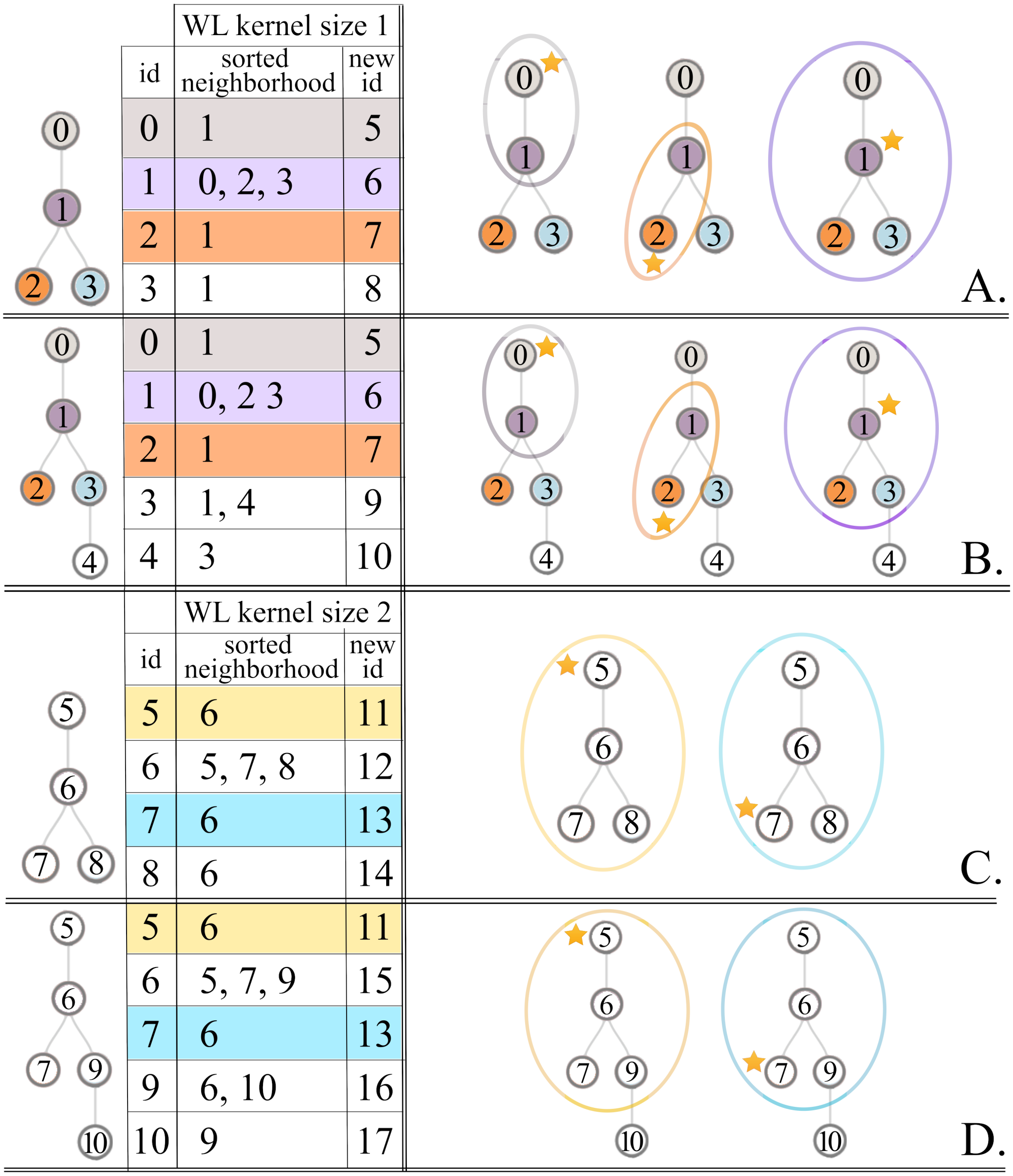

	WL kernel size 1	
id	sorted neighborhood	new id
0	1	5
1	0, 2, 3	6
2	1	7
3	1	8
0	1	5
1	0, 2 3	6
2	1	7
3	1, 4	9
4	3	10

	WL kernel size 2	
id	sorted neighborhood	new id
5	6	11
6	5, 7, 8	12
7	6	13
8	6	14
5	6	11
6	5, 7, 9	15
7	6	13
9	6, 10	16
10	9	17

Supplementary Figure 1: Example of neighborhood matching scheme between two trees using the Weisfeiler-Lehman (WL) kernel. (A,B) For each of the two trees, each node is represented as a sorted list of its first degree neighbors and an id is assigned to each such neighborhood. The new ids encode the neighborhoods of size 1 around each node. There are 3 matching neighborhoods of size 1 between the two trees, around the nodes indicated with stars. Note that a neighborhood of size 1 around the middle node of the first tree covers the entire tree. **(C,D)** A new iteration of the WL subtree kernel is equivalent to encoding neighborhoods of size 2 around every node. The trees are relabeled with the new ids computed in the previous iteration. There are 2 matching neighborhoods of size 1 between the two trees, around the nodes indicated with stars. The colors help matching the tree neighborhood visualization with the corresponding neighborhood labels from the table.

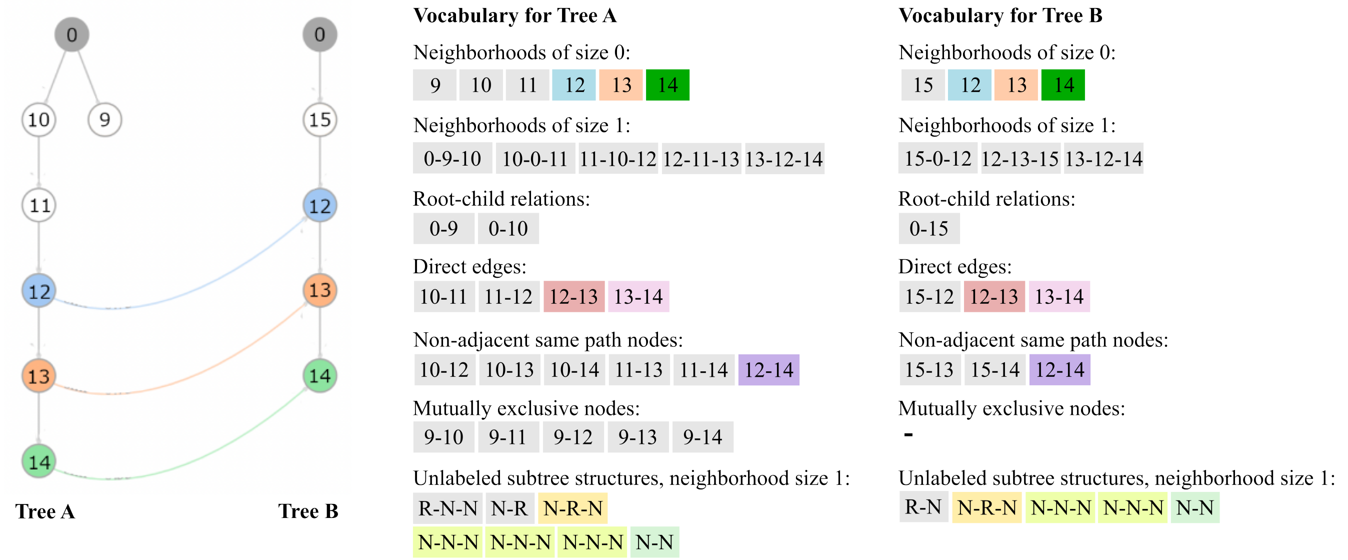

Supplementary Figure 2: Example of vocabulary construction and feature matching between two labeled trees. The tree nodes have numeric labels (e.g., that encode genes with point mutations). The vocabulary of each tree for all the categories of node relations, as described in Section 2.1, is shown. The vocabulary "words" are concatenations of node labels, which are further used in our implementation as hash compressed strings. The matching node relation patters are highlighted with colors, while non-matching ones are gray. Redundant relations are only added ones, i.e., the direct edges are excluded from the neighborhoods and the root-child relations are excluded from the direct edges. For the unlabeled tree structures "N" represents internal nodes and "R" represents the root. An algorithmic perspective for the vocabulary construction is shown in Suppl. Algorithm 1.

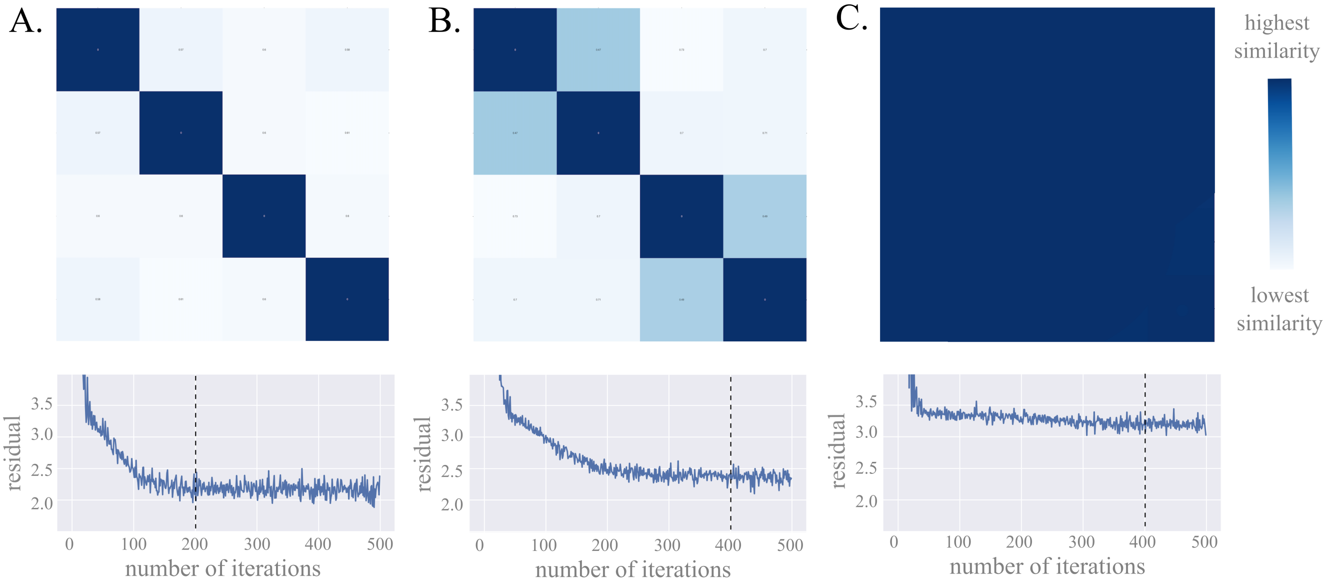

Supplementary Figure 3: Heatmap visualization of tree similarities for small tree cohorts (4 trees) in 3 scenarios: **(A)** 4 unrelated trees (no match); **(B)** 2 pairs of matching trees; **(C)** 4 identical trees. The convergence plot is shown below each heatmap. The dotted lines shows where the convergence plots become steady, i.e., the training algorithm starts to converge (this is the cutoff used).

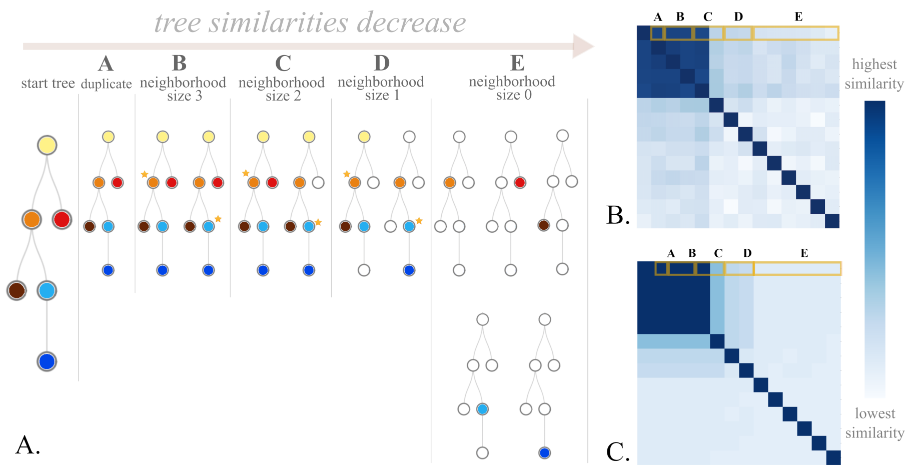

D.

category	number of vocabulary intersections	rank	similarity w.r.t. start tree	minimum similarity in previous rank	similarity deviation from expected rank
A	114	1	0.9728	0.9728	0
B	114	1	0.9719	0.9728	0
B	114	1	0.9702	0.9728	0
C	114	1	0.9667	0.9728	0
C	67	2	0.7313	0.9667	0
D	61	3	0.7182	0.7313	0
D	60	4	0.7198	0.7182	1.5e-3
E	55	5	0.6895	0.7198	0
E	55	5	0.6837	0.7186	0
E	55	5	0.6823	0.7186	0
E	55	5	0.6802	0.7186	0
E	55	5	0.6690	0.7186	0
E	55	5	0.6514	0.7186	0

Supplementary Figure 4: Example of simulated mutation trees with decreasing similarities, starting from a given start tree. **(A)** Tree group construction: all trees share the same tree structure, but the number of matching nodes is restricted to specific neighborhoods of decreasing sizes around every node with degree greater than 2 (for example, the starred nodes). Matching nodes are colored the same. In this particular example, a neighborhood of size 3 around a middle node covers the whole tree, leading to an identical match. Nevertheless, a duplicate of the start tree is included in the small cohort from the beginning. The categories A-E denote the different matching neighborhood sizes and the similarities w.r.t. the start tree are expected to decrease as the size of the matching neighborhoods decreases. **(B)** Corresponding tree distance heatmap based on the embeddings learned by oncotree2vec. The heatmap is built using hierarchical clustering with the cosine distance metric on the tree embeddings. **(C)** Heatmap showing the number of shared vocabulary words (encoding neighborhoods of different sizes) for each pair of trees. **(D)** Similarity of each tree w.r.t. the start tree using the learned embeddings (highest similarity is 1). The similarity scores are ordered by the number of vocabulary intersections, in decreasing order. The maximum intersection size, 144, corresponds to the identical match to the start tree and is computed as the number of tree nodes times the size of the largest WL kernel applied for the labeled neighborhoods and subtree structures, i.e., $6x(10+9)$ – see also Section 2.1. Similarity scores corresponding to the same rank are colored the same. Each similarity score should be lower or equal to the minimum score from the previous rank. The deviation of the embedding similarities from the expected order is computed by subtracting, for each tree, the computed similarity w.r.t. the start tree from the minimum similarity score from the previous rank. In this example, there is one embedding deviating from the expected ordering (similarity deviation 0.0015). The average deviation from the expected rank ordering is 0.000782, which is very close to zero, the deviation for perfect ordering.

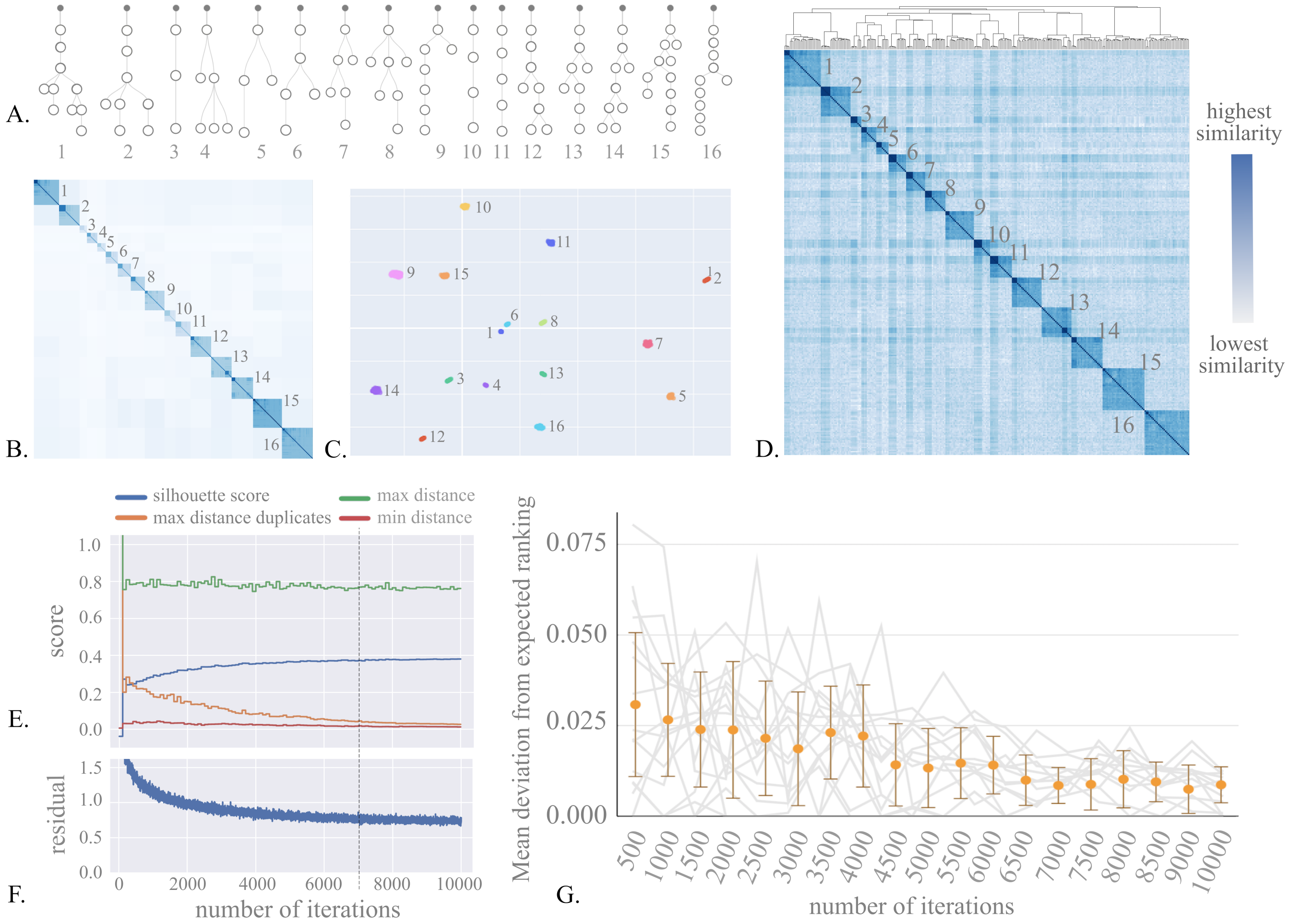

Supplementary Figure 5: Clustering a cohort of 16 groups of synthetic trees (synthetic dataset I). (A) Tree structures of the start trees for each of the 16 groups. **(B)** Heatmap showing the number of elements of the vocabulary intersections for each pair of trees. White color corresponds to empty intersections, and dark blue indicates large vocabulary intersections. **(C)** UMAP showing the separation between the inferred tree clusters using the cosine distance between the learned embeddings. **(D)** Heatmap visualization of tree similarities for the simulated trees from 16 groups, using the cosine distance between the embeddings learned by oncotree2vec. The trees cluster by simulation group. In panels B-D the numbers correspond to the tree indices in panel A and indicate the structures of the trees in each group. **(E)** Minimum and maximum cosine distance between the learned embeddings after every training iteration (see Section 2.2). **(F)** The residual after each training iteration. The dotted line shows where the convergence plot becomes steady, i.e., the training algorithm starts to converge (this is the cutoff used). **(G)** Line chart showing the deviation from the expected rank ordering of the trees in each group. The lines correspond to the scores computed after every 5,000 iterations (10,000 iterations in total). The error bars show the average deviation from the expected rank ordering across all the 16 cohorts and the corresponding standard deviation.

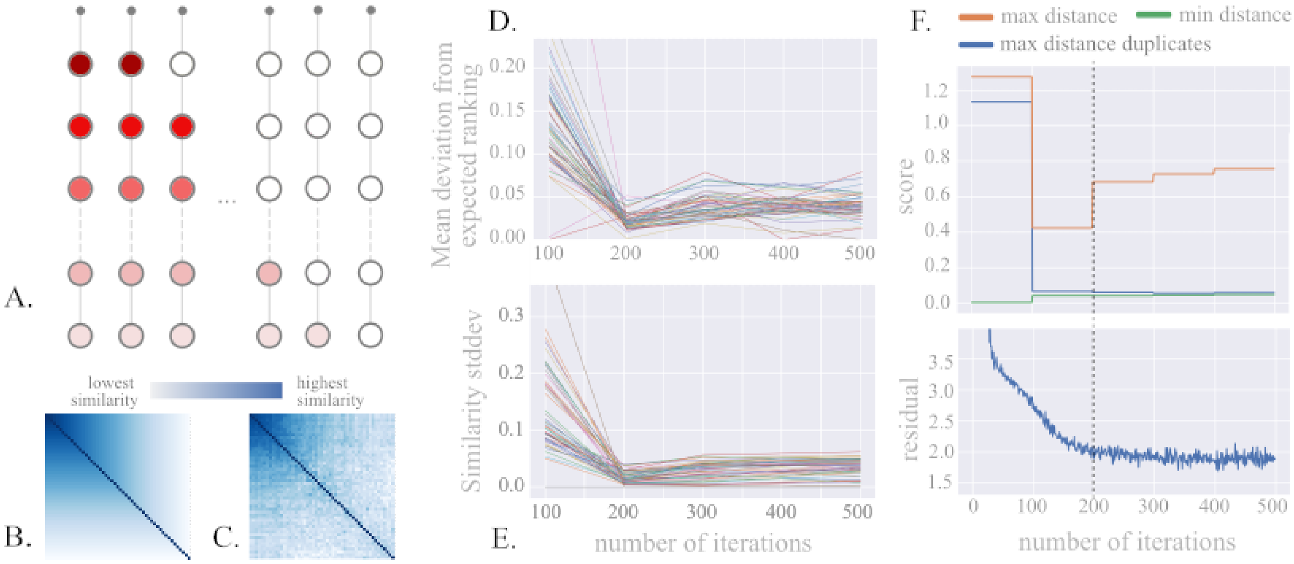

Supplementary Figure 6: Clustering synthetic trees with known similarity rank ordering (synthetic dataset II). (A) Cohort of synthetic linear trees with decreasing number of matching nodes. Matching nodes are colored the same. **(B)** Heatmap showing the number of matching nodes for each pair of trees, sorted by the number of matches. This is the expected tree rank ordering. Note that the trees from the antidiagonals parallel to the main antidiagonal share the same number of matching nodes (the pairwise vocabulary intersections have the same number of elements). **(C)** Tree similarity heatmap of the pairwise distances between the simulated trees, using the cosine distance between the learned embeddings. The order of the trees corresponds to the one in panel B. **(D)** Line chart showing the deviation of the embedding similarities from the expected rank ordering for all tree in the cohort (average value), computed every 100 iterations. **(E)** Standard deviation of the pairwise tree embedding distances between the learned tree embeddings corresponding to pairs of trees with the same number of matching nodes, computed every 100 iterations. Each line corresponds to the pairs of trees that share a number of matching nodes ranging from 0 to 50, which appear on the same antidiagonal in panel B. **(F)** Minimum and maximum cosine distance between the learned embeddings (see Methods 2.2) and the residual after each training iteration. The dotted line shows where the convergence plot becomes steady, i.e., the training algorithm starts to converge (this is the cutoff used).

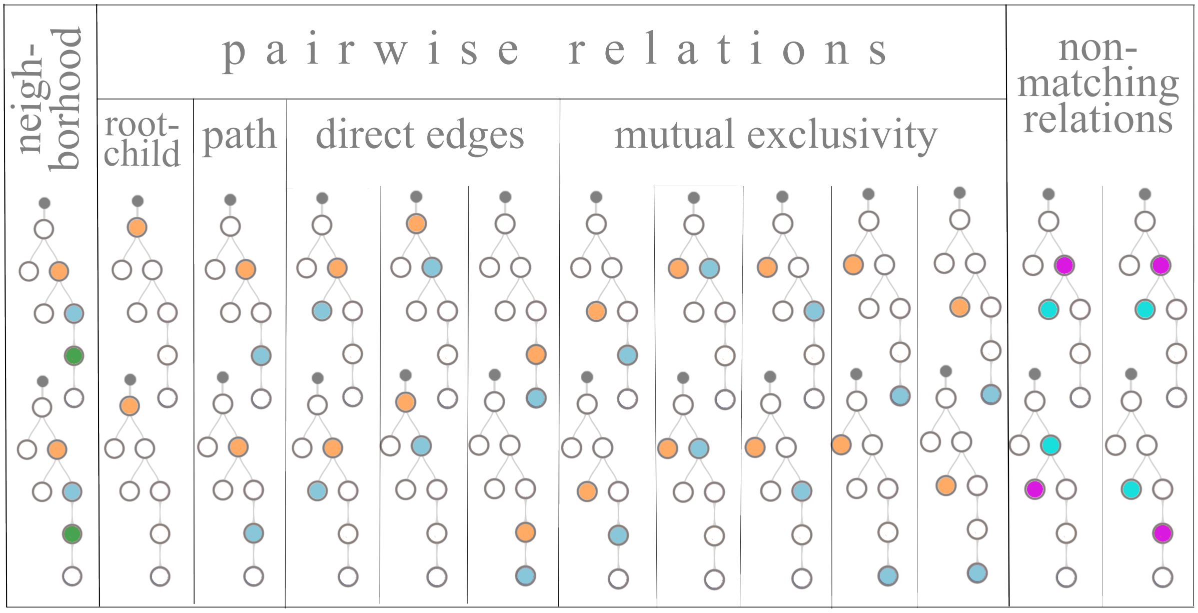

Supplementary Figure 7: Matching and non-matching pairs of trees used in synthetic dataset III. The colored nodes correspond to nodes with matching labels, while the white nodes do not match (they are labelled differently). Each pair of matching trees contains one precise matching pattern from each vocabulary category, as described in Section 2.1. The two non-matching pairs of trees highlight cases where some individual nodes match, but their order is reversed, or they belong to different branches – these are considered non-matching node pairwise relations because they correspond to different tumor evolutions.

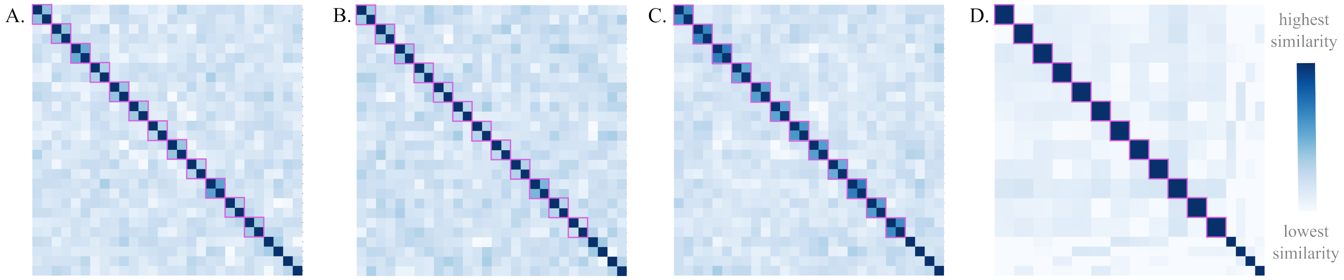

Supplementary Figure 8: Heatmap visualization of tree similarities for the selected pairs of trees in synthetic dataset III, using graph2vec and oncotree2vec with different parameters: **(A)** graph2vec – WL kernel size 1, **(B)** graph2vec – WL kernel size 2, **(C)** oncotree2vec – WL kernel size 0 (without neighborhoods) **(D)** oncotree2vec – WL kernel size 1. The violet rectangles indicate the expected matching pairs. All the other pairs (between trees which are not expected to match) are referred to as "negatives". The similarity scores encoded by the pixels corresponding to the matching pairs are listed in Table 1 of the main paper (last four rows).

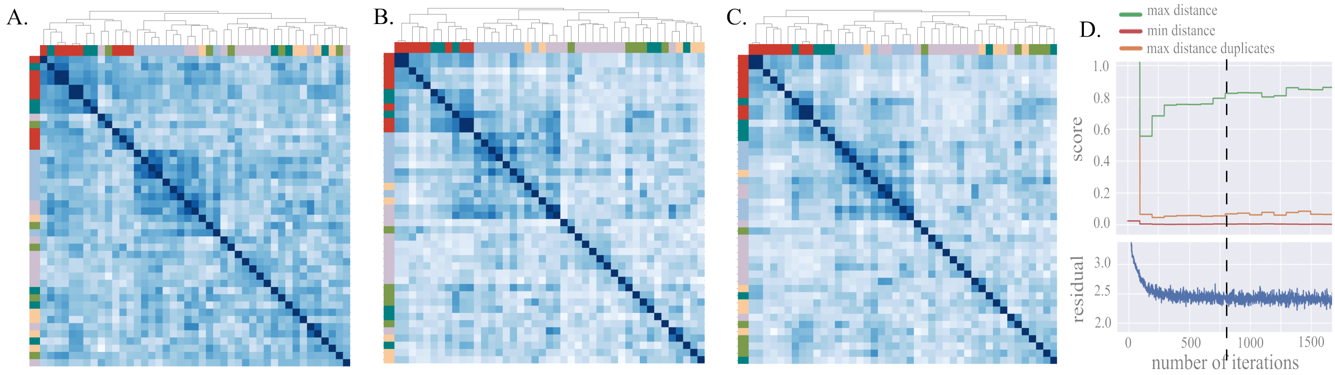

Supplementary Figure 9: Result of clustering tree structures from six cancer types from Noble et al. 2022 using embedding sizes of 32 **(A)**, 64 **(B)** and 128 **(C)**. **(D)** Training information for embedding size of 64: minimum and maximum cosine distance between the learned embeddings, the maximum cosine distance between duplicated trees (see Section 2.2) and the residual after each training iteration. The dotted line shows where the convergence plot becomes steady, i.e., the training algorithm starts to converge (this is the cutoff used).

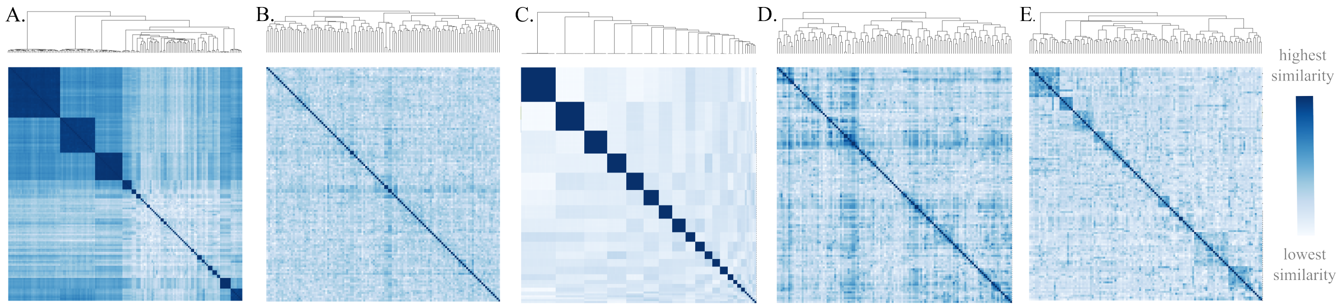

Supplementary Figure 10: Hierarchically-clustered heatmap of tree similarities for the AML mutation trees from Morita et al. 2020 using different vocabulary augmentation amounts. **(A)** vocabulary contains only on the tree structures (the labels are discarded); **(B)** vocabulary contains neighborhoods of all sizes up to size 3 – this would be the result obtained using graph2vec; **(C)** vocabulary contains only root child relations; **(D)** vocabulary contains individual nodes, pairwise relations and mutually exclusive pairs; **(E)** vocabulary is based on all the subtree structure categories described in Fig. 2 of the main paper, with the category of pairwise-relations over-represented (augmented).

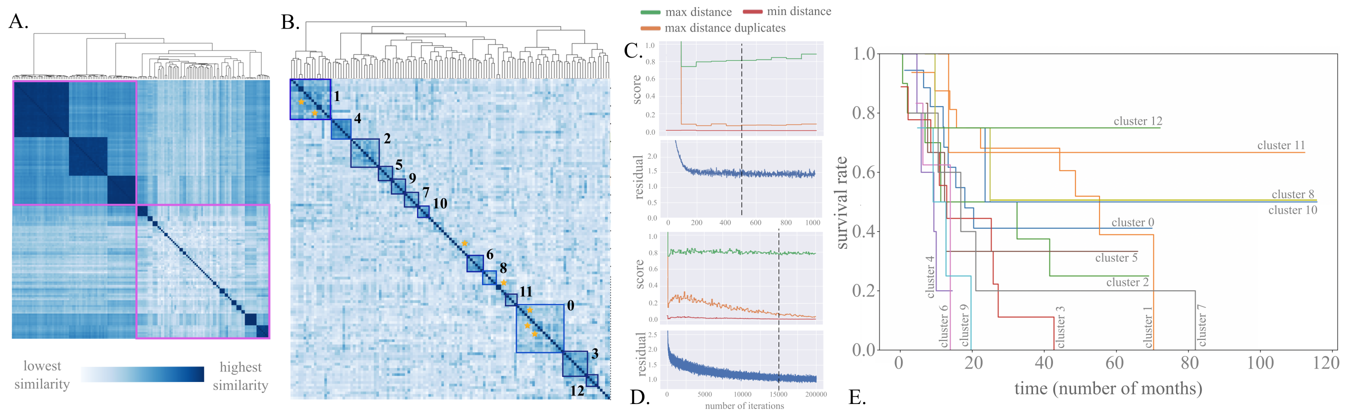

Supplementary Figure 11: Clustering results for a cohort of 123 AML mutation trees. (A) Heatmap visualization of tree similarities based on the tree structures. The cluster split (the violet rectangles) indicates two different modes of clonal evolution: linear and branching. **(B)** Heatmap visualization of tree similarities based on the individual sub-clone labels and different labeling relations: neighborhoods of different sizes, root-child relations, direct edges, non-adjacent same path pairwise relations and mutually exclusive relations. The large clusters are indicated by squares, and small clusters are highlighted by a star. **(C, D)** Training information for the experiments in panels A and B, respectively: minimum and maximum cosine distance between the learned embeddings, the maximum cosine distance between duplicated trees (see Methods 2.2) and the residual after each training iteration. The dotted line shows where the convergence plot becomes steady and the training algorithm starts to converge (this is the cutoff used). **(E)** Kaplan Meier curve estimation of the survival for 13 clusters found in the AML mutation tree cohort (Section 3.3). The cluster numbers in panels B and E correspond to the ones in Suppl. Table 3.

Supplementary Figure 12: Visualization of the AML mutation tree clusters obtained using oncotree2vec. Matching nodes are colored the same.

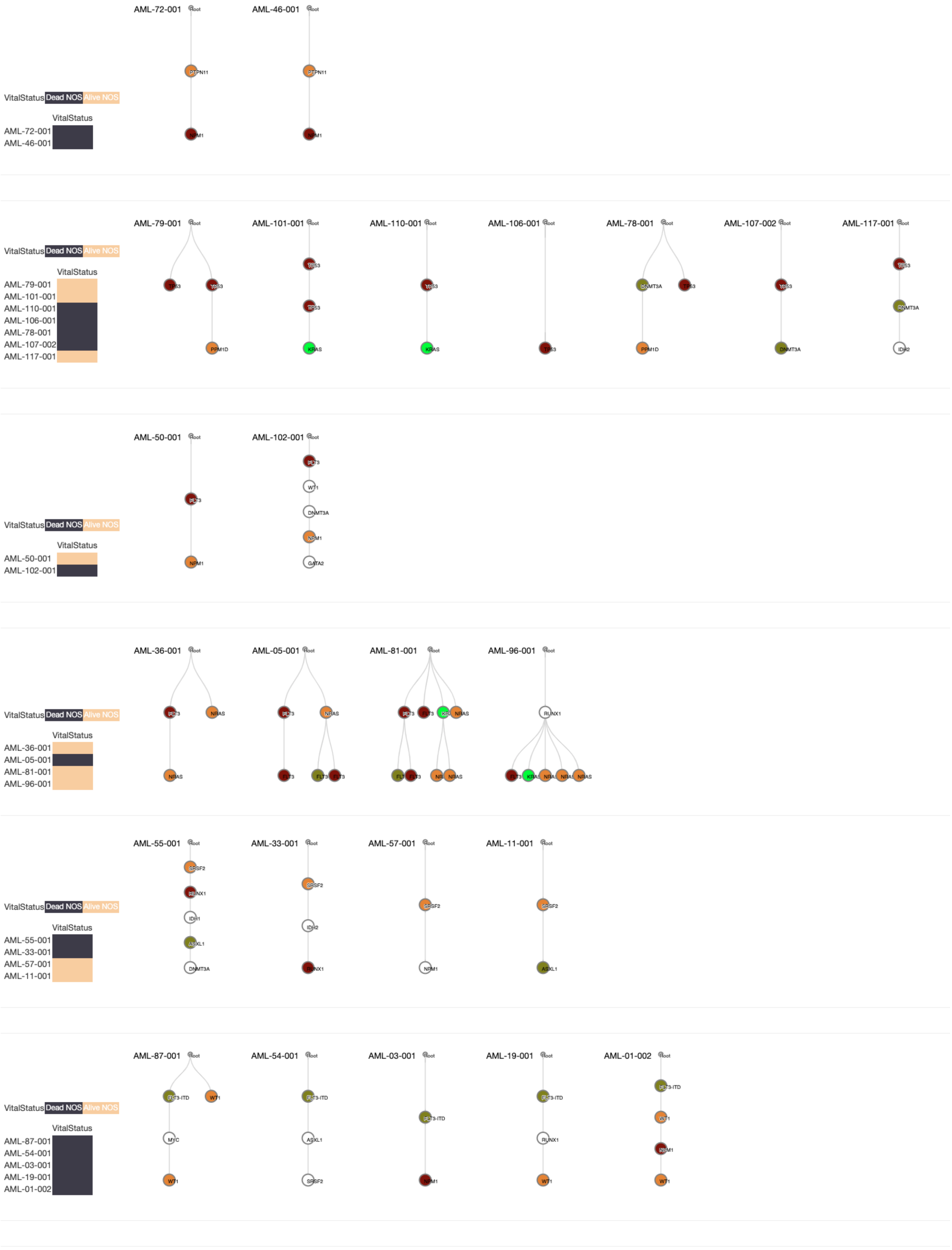

VitalStatus Dead NOS Alive NOS
VitalStatus
AML-72-001
AML-46-001
VitalStatus Dead NOS Alive NOS
VitalStatus
AML-79-001
AML-101-001
AML-110-001
AML-106-001
AML-78-001
AML-107-002
AML-117-001
VitalStatus Dead NOS Alive NOS
VitalStatus
AML-50-001
AML-102-001
VitalStatus Dead NOS Alive NOS
VitalStatus
AML-36-001
AML-05-001
AML-81-001
AML-96-001
VitalStatus Dead NOS Alive NOS
VitalStatus
AML-55-001
AML-33-001
AML-57-001
AML-11-001
VitalStatus Dead NOS Alive NOS
VitalStatus
AML-87-001
AML-54-001
AML-03-001
AML-19-001
AML-01-002

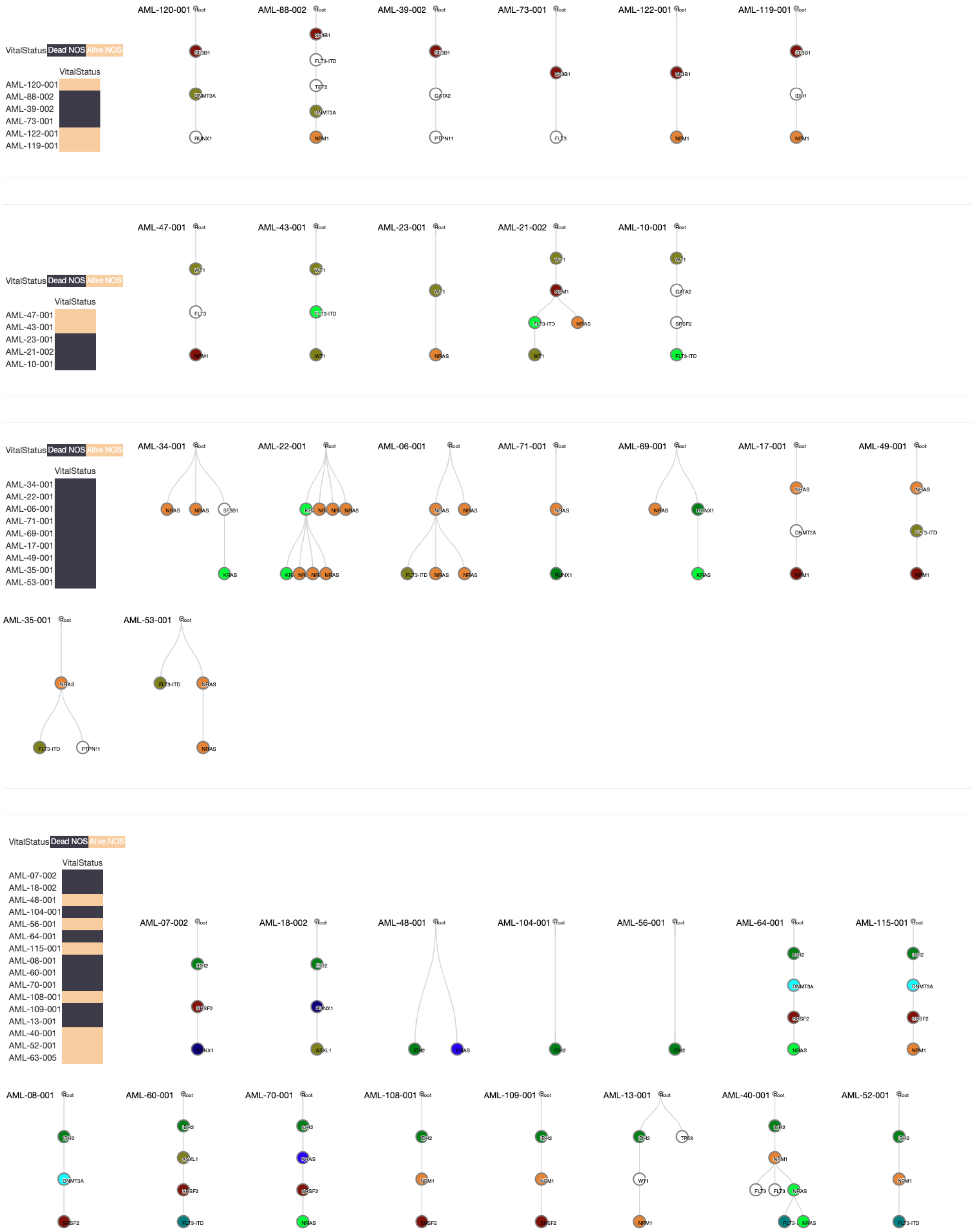

VitalStatus Dead NOS Alive NOS
VitalStatus
AML-120-001
AML-88-002
AML-39-002
AML-73-001
AML-122-001
AML-119-001
AML-47-001
AML-43-001
AML-23-001
AML-21-002
AML-10-001
AML-34-001
AML-22-001
AML-06-001
AML-71-001
AML-69-001
AML-17-001
AML-49-001
AML-35-001
AML-53-001
AML-07-002
AML-18-002
AML-48-001
AML-104-001
AML-56-001
AML-64-001
AML-115-001
AML-08-001
AML-60-001
AML-70-001
AML-108-001
AML-109-001
AML-13-001
AML-40-001
AML-52-001
AML-63-005

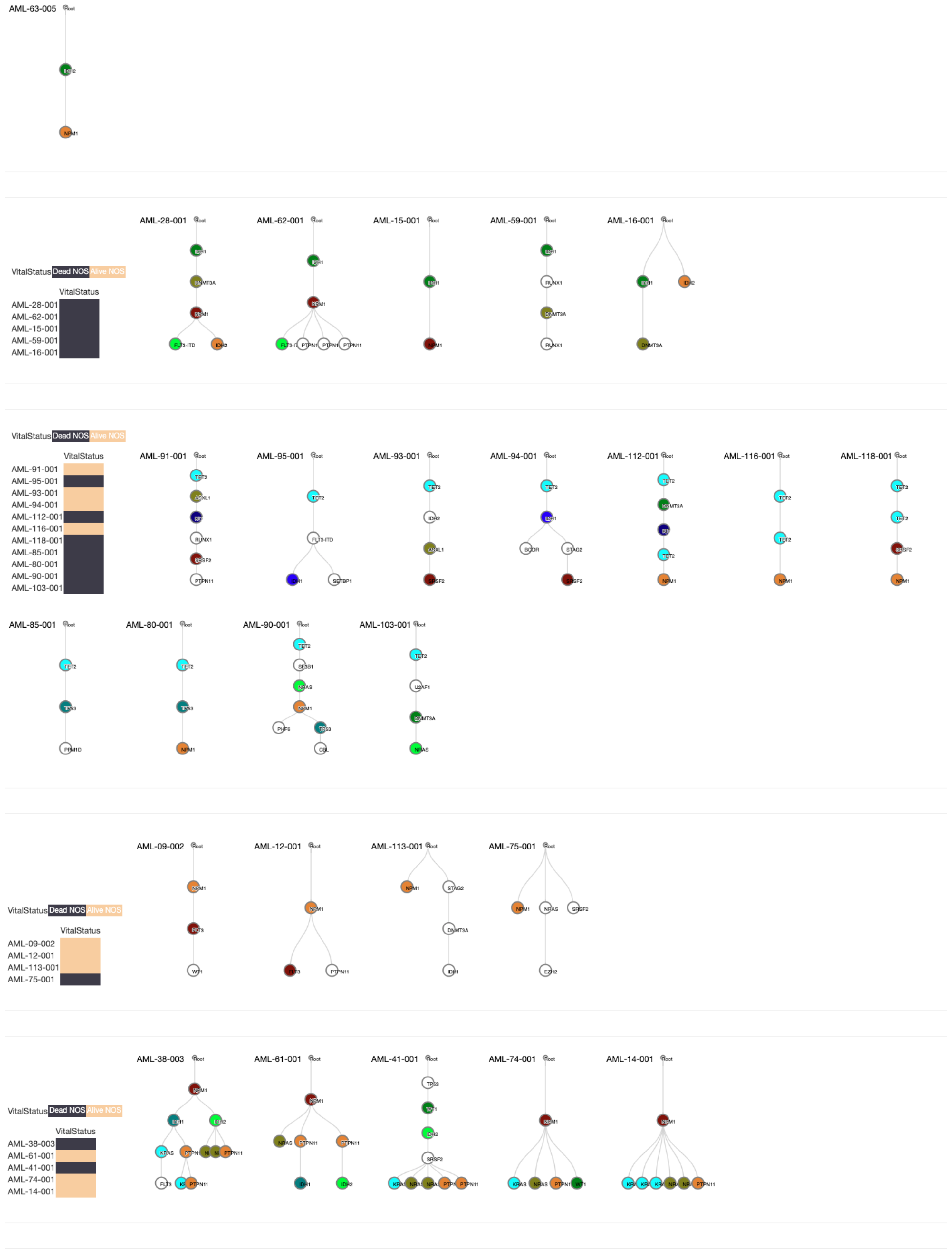

AML-63-005
VitalStatus Dead NOS Alive NOS
VitalStatus
AML-28-001
AML-62-001
AML-15-001
AML-59-001
AML-16-001
AML-91-001
AML-95-001
AML-93-001
AML-94-001
AML-112-001
AML-116-001
AML-118-001
AML-85-001
AML-80-001
AML-90-001
AML-103-001
AML-09-002
AML-12-001
AML-113-001
AML-75-001
AML-38-003
AML-61-001
AML-41-001
AML-74-001
AML-14-001

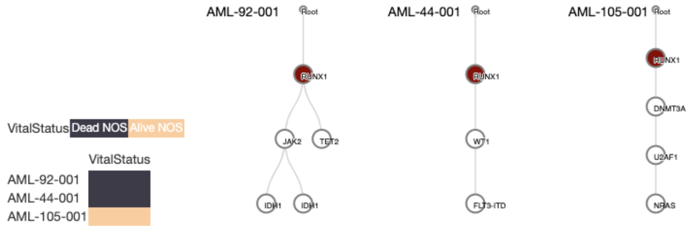
VitalStatus Dead NOS Alive NOS
VitalStatus
AML-92-001
AML-44-001
AML-105-001
AML-92-001
AML-44-001
AML-105-001

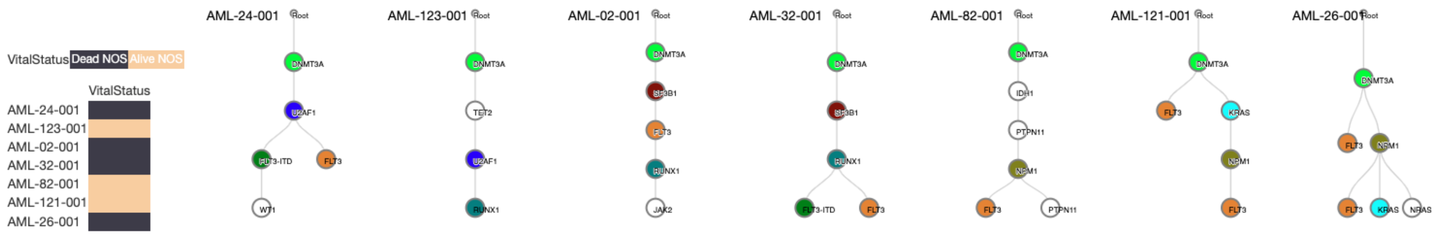
VitalStatus Dead NOS Alive NOS
VitalStatus
AML-24-001
AML-123-001
AML-02-001
AML-32-001
AML-82-001
AML-121-001
AML-26-001
AML-24-001
AML-123-001
AML-02-001
AML-32-001
AML-82-001
AML-121-001
AML-26-001

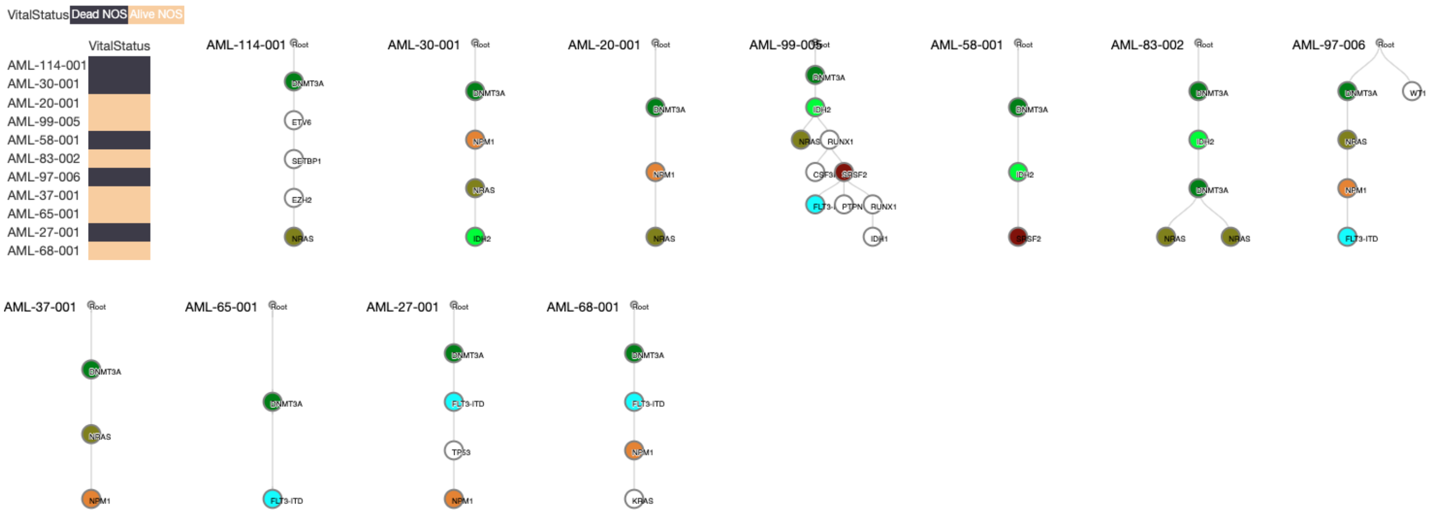
VitalStatus Dead NOS Alive NOS
VitalStatus
AML-114-001
AML-30-001
AML-20-001
AML-99-005
AML-58-001
AML-83-002
AML-97-006
AML-37-001
AML-65-001
AML-27-001
AML-68-001
AML-114-001
AML-30-001
AML-20-001
AML-99-005
AML-58-001
AML-83-002
AML-97-006
AML-37-001
AML-65-001
AML-27-001
AML-68-001

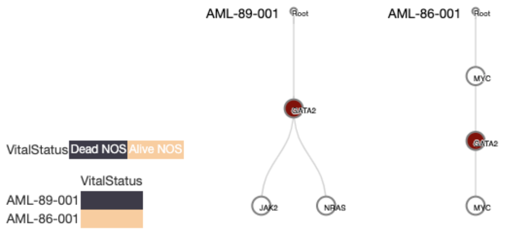
AML-89-001
AML-86-001
VitalStatus Dead NOS Alive NOS
VitalStatus
AML-89-001
AML-86-001

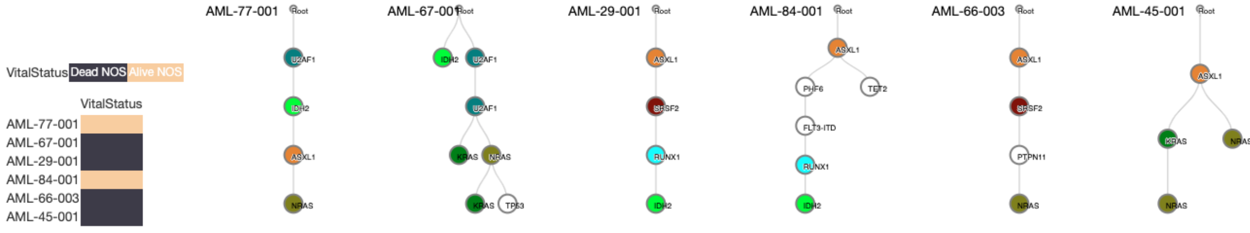
AML-77-001
AML-67-001
AML-29-001
AML-84-001
AML-66-003
AML-45-001
VitalStatus Dead NOS Alive NOS
VitalStatus
AML-77-001
AML-67-001
AML-29-001
AML-84-001
AML-66-003
AML-45-001

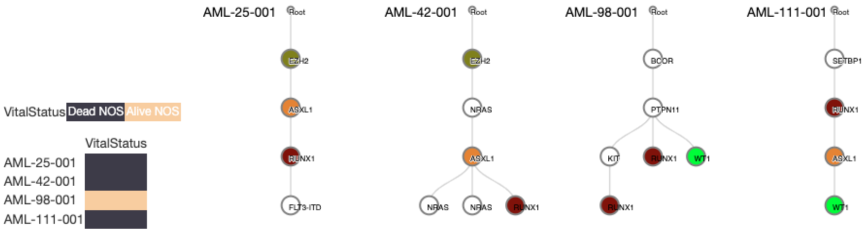
VitalStatus
Dead NOS
Alive NOS
VitalStatus
AML-25-001
AML-42-001
AML-98-001
AML-111-001
AML-25-001
Root
EZH2
ASXL1
RUNX1
FLT3-ITD
AML-42-001
Root
EZH2
NRAS
ASXL1
NRAS
NRAS
RUNX1
AML-98-001
Root
BCOR
PTPN11
KIT
RUNX1
WT1
RUNX1
AML-111-001
Root
SETBP1
RUNX1
ASXL1
WT1

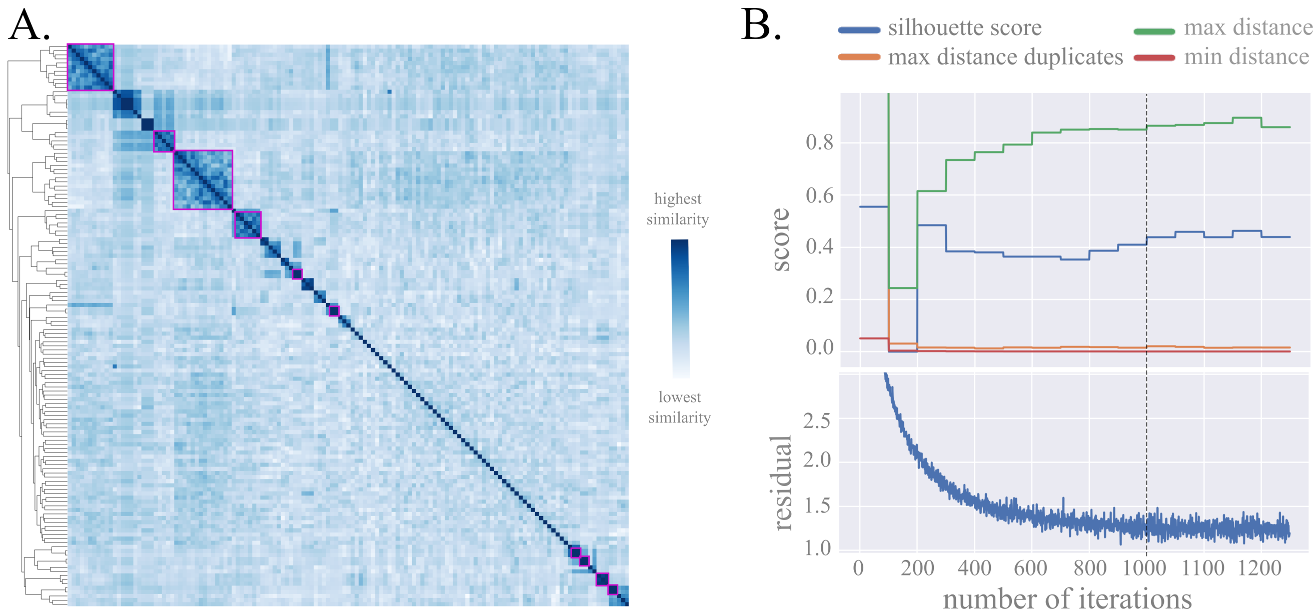

Supplementary Figure 13: Experiment to assess the robustness of oncotree2vec when handling the uncertainty output by the tree inference tools. (A) Result of clustering 137 tumor mutation trees (99 patients) inferred with REVOLVER (Caravagna *et al.*, 2018) from the TRACERx NSCLC non-small-cell lung cancer multi-region whole-exome sequencing data (Jamal-Hanjani *et al.*, 2017), using a maximum likelihood approach. REVOLVER reported multiple mutation trees with high likelihood for 10 of the patients, and the most likely reconstructed tree for the rest of the patients. Using oncotree2vec we learned embeddings that encode the individual subclones and the pairwise relations between subclones (the complete set of parameters used for training are available in Suppl. Table 2). As expected, the resulting clustering of the mutation trees in the embedding space reflects the different posterior distributions. This is due to a larger vocabulary intersection between the mutation trees that belong to the same posterior distribution, which share the same subclones and similar clonal lineages, compared to the rest of the trees. The trees which correspond to the same posterior distribution cluster together and are highlighted by violet squares on the heatmap. Each highlighted cluster corresponds to the mutation trees inferred for each of the 10 patients mentioned before: CRUK0001 (11 trees), CRUK0013 (4 trees), CRUK0016 (14 trees), CRUK0063 (6 trees), CRUK0062 (2 trees), CRUK0002 (2 trees), CRUK0020 (2 trees), CRUK0024 (2 trees), CRUK0006 (3 trees), CRUK0023 (2 trees). Additional weaker similarities were encountered between some mutation tree pairs from patients (from different posterior distributions) that share one subclone mutation. We evaluated the separation between the 10 clusters of trees which belong to different posterior distributions (all the other trees are discarded) and obtained a silhouette score of 0.63, which indicates a good cluster separation. **(B)** Minimum and maximum cosine distance between the learned embeddings (see Methods 2.2) and the residual after each training iteration. The dotted line shows where the convergence plot becomes steady, i.e., the training algorithm starts to converge (cutoff for the optimal solution at 1,000 iterations). The overall silhouette score for the optimal solution (all the trees included) is 0.4.

References

Caravagna G, Giarratano Y, Ramazzotti D, *et al.* Detecting repeated cancer evolution from multiregion tumor sequencing data. *Nature Methods* **15**(9):707–714, 2018.

Jamal-Hanjani M, Wilson GA, McGranahan N, *et al.* Tracking the evolution of non-small-cell lung cancer. *The New England Journal of Medicine* **376**(22):2109–2121, 2017.
